# Clinical, Economic, and System-Level Outcomes of Bariatric Surgery in Adults: Protocol for a Systematic Review

**DOI:** 10.2196/98976

**Published:** 2026-10-19

**Authors:** Michał Bystram, Marlena Robakowska, Monika Proczko-Stepaniak

**Affiliations:** 1 Department of Surgical Oncology, Transplant Surgery and General Surgery, Gdańsk Medical University, Marii Skłodowskiej-Curie 3a, Gdansk, Pomerania, 80210, Poland, 48 519839113; 2 Division of Public Health & Social Medicine, Gdańsk Medical University, Gdansk, Pomerania, Poland

**Keywords:** bariatric surgery, cost-effectiveness, health care costs, resource utilization, systematic review

## Abstract

**Background:**

Obesity affects approximately 880 million adults worldwide and carries substantial health, economic, and social consequences. Although bariatric surgery achieves sustained weight loss and metabolic improvement, existing systematic reviews largely predate 2020 and rarely integrate clinical, economic, and system-level perspectives within a single synthesis.

**Objective:**

This study aims to identify, critically appraise, and synthesize evidence published between 2020 and 2025 on the clinical effectiveness and economic impact of bariatric surgery in adults with obesity. A secondary, exploratory objective is to address system-level resource utilization, specifically length of stay, preoperative pathway duration, and outpatient care burden.

**Methods:**

This protocol follows the PRISMA-P (Preferred Reporting Items for Systematic Review and Meta-Analysis Protocols) 2015 guidance and is registered with the International Prospective Register of Systematic Reviews (PROSPERO: CRD420261351022). Eligible studies will enroll adults aged 18 years or older with obesity (BMI ≥30 kg/m^2^) undergoing any primary bariatric procedure, including Roux-en-Y gastric bypass (RYGB), sleeve gastrectomy (SG), one-anastomosis gastric bypass (OAGB), adjustable gastric banding (LAGB), and biliopancreatic diversion with or without duodenal switch (BPD±DS); revisional procedures are excluded as the index intervention. Eligible designs include randomized controlled trials, cohort, case-control, cross-sectional, and economic evaluation studies published in English. MEDLINE (via PubMed), Embase, Scopus, and Web of Science will be searched using predefined, database-tailored strategies; records will be managed in Zotero (Corporation for Digital Scholarship). A single primary reviewer will perform screening, extraction, and risk-of-bias (RoB) assessment, with at least 20% independently duplicated by a second reviewer, weighted toward full-text and included studies. RoB will be assessed with RoB 2 and ROBINS-I (Risk of Bias in Nonrandomized Studies of Interventions); economic evaluations will be appraised for completeness of reporting with the CHEERS (Consolidated Health Economic Evaluation Reporting Standards) 2022 statement and for methodological quality with the CHEC (Consensus on Health Economic Criteria) checklist. The primary synthesis will be narrative; meta-analysis will be considered only where prespecified comparability criteria are met. Certainty of evidence will be rated using GRADE (Grading of Recommendations Assessment, Development, and Evaluation).

**Results:**

This protocol describes a review that is currently in progress. Registration with PROSPERO was completed on April 15, 2026, and database searches were run on August 1, 2026, identifying 9400 records. Title-and-abstract screening is scheduled to begin on August 15, 2026. Data extraction and synthesis are expected to be completed by the end of 2026, with publication of the completed review anticipated in the first half of 2027.

**Conclusions:**

This protocol provides a transparent and reproducible methodology for an integrated, contemporary synthesis of the clinical, economic, and system-level outcomes of bariatric surgery in adults. The findings are intended to inform health policy, resource planning, and future research priorities.

## Introduction

### Burden of Obesity

Obesity is a chronic and multifactorial disease defined by excessive accumulation of body fat that is associated with significant health risks [1,2]. Between 1990 and 2022, the global prevalence of obesity among adults more than doubled in women and nearly tripled in men, and by 2022, an estimated 880 million adults had a BMI of 30 kg/m^2^ or higher [3]. Obesity increases the risk of disease and premature death and can lead to a range of health problems, including type 2 diabetes, heart disease, hypertension, dyslipidemia, sleep apnea, fatty liver disease, osteoarthritis, and certain cancers [4,5]. Its consequences extend beyond personal health, generating higher health care costs related to both the treatment of associated diseases and lost productivity, disability, and premature death [6]. People with obesity also frequently experience stigmatization and discrimination that can affect their quality of life [7]. Although lifestyle interventions and pharmacological therapies are available, many people with obesity do not achieve or maintain clinically significant weight loss using nonsurgical methods alone [8], underscoring the need for effective, evidence-based treatments that achieve sustained weight loss and metabolic benefits.

### Role of Bariatric Surgery

Bariatric surgery encompasses several procedures that promote weight loss by restricting food intake, reducing nutrient absorption, or combining both mechanisms. Commonly performed procedures include Roux-en-Y gastric bypass (RYGB), sleeve gastrectomy (SG), one-anastomosis gastric bypass (OAGB), adjustable gastric banding (LAGB), and biliopancreatic diversion with or without duodenal switch (BPD±DS) [9]. Extensive evidence indicates that bariatric surgery is an effective method for achieving significant and sustained weight loss in people with obesity [10-12]. In addition to weight reduction, bariatric surgery is associated with remission or improvement of obesity-related comorbidities, particularly type 2 diabetes, hypertension, dyslipidemia, and obstructive sleep apnea [13,14]. Long-term studies further indicate improved cardiovascular outcomes, reduced cancer incidence, and reduced all-cause mortality compared with nonsurgical treatment [15,16]. The mechanisms responsible for these benefits are multifaceted and extend beyond calorie restriction: bariatric procedures modify the anatomy and physiology of the gastrointestinal tract, leading to changes in the secretion of intestinal hormones (including ghrelin, glucagon-like peptide 1, and peptide YY), bile acid metabolism, gut microbiota composition, and energy expenditure [17,18]. These adaptations contribute to improved glycemic control, increased insulin sensitivity, and an improved lipid profile, often preceding significant weight loss [19].

### Clinical, Economic, and System-Level Relevance

With the growing adoption of bariatric surgery, there is increasing recognition that a comprehensive assessment of its effects should extend beyond traditional clinical outcomes, because health care decision-makers, policymakers, and payers require evidence across 3 interrelated domains. Clinical outcomes are fundamental to assessing surgical effectiveness; key indicators include the magnitude and durability of weight loss, expressed as percentage total weight loss (%TWL) as the primary metric consistent with contemporary reporting standards [20], indicators of metabolic remission, the incidence and severity of postoperative complications (often classified using the Clavien-Dindo system [21]), reoperation rates, and short- and long-term mortality. Assessing the safety and efficacy profiles of different procedures is essential for patient counseling, surgical decision-making, and quality assurance [22].

Economic outcomes are critical for resource allocation and health policy. Direct health care costs associated with bariatric surgery include hospitalization, surgery, treatment of postoperative complications and readmissions, outpatient visits, pharmacotherapy, and diagnostics [23,24]. Beyond direct costs, economic evaluations assess cost-effectiveness using measures such as the incremental cost-effectiveness ratio (ICER), cost-utility analyses incorporating quality-adjusted life years (QALYs), net monetary benefit, and budget impact analyses that estimate the annual cost of implementing bariatric surgery programs [25,26]. Evaluating the economic value of bariatric surgery relative to alternative interventions or standard care is essential for informed health care investment decisions [27]. Finally, system-level resource utilization reflects the impact of bariatric surgery on health care delivery. Key indicators include length of stay, readmission rates, time from surgical qualification to surgery (preoperative pathway duration), outpatient care burden, and implementation of perioperative care pathways, such as enhanced recovery after surgery protocols [28]. These outcomes are essential for capacity planning, workflow optimization, and understanding the broader implications of expanding bariatric surgery programs [29].

### Rationale for the Review

Existing systematic reviews of bariatric surgery outcomes largely reflect data collected before 2020, with few reviews covering the period 2020 to 2025 and few considering clinical, economic, and system-level perspectives together. In the context of evolving surgical practice, increasing patient case complexity, and changing health care financing, an updated and integrated synthesis is needed. A protocol-driven approach is fundamental for ensuring transparency, methodological rigor, and reproducibility. The present protocol follows the PRISMA-P (Preferred Reporting Items for Systematic Review and Meta-Analysis Protocols) guidance (Checklist 1) [30] and will direct the systematic review in accordance with PRISMA (Preferred Reporting Items for Systematic Review and Meta-Analyses) 2020 standards [31].

### Objectives and Review Questions

The primary objective is to systematically identify, critically appraise, and synthesize contemporary evidence (2020‐2025) on the clinical effectiveness and economic impact of bariatric (metabolic) surgery in adults aged 18 years or older with obesity (BMI≥30 kg/m^2^), compared with nonsurgical management or alternative surgical procedures, with respect to weight loss, metabolic remission, complications, health care costs, and cost-effectiveness. As a secondary, exploratory objective, the review will describe system-level resource utilization associated with bariatric surgery, specifically length of stay, preoperative pathway duration, outpatient care burden, and readmission. This domain is designated exploratory because the relevant indicators are defined inconsistently across health care systems and are reported less systematically than clinical and economic outcomes; findings will therefore be presented descriptively and will not be assigned priority-outcome status in the synthesis or in the GRADE (Grading of Recommendations Assessment, Development, and Evaluation) assessment.

The review will address 3 questions. First, what are the clinical outcomes (weight loss, metabolic remission, complications, reoperations, and mortality) of bariatric surgery in adults with obesity? Second, what are the economic outcomes (direct health care costs, cost-effectiveness, cost-utility, and budget impact) associated with bariatric surgery? Third, as an exploratory question, what is the system-level resource utilization (length of stay, readmission rates, preoperative pathway length, and outpatient care burden) associated with bariatric surgery? Throughout, the 3 outcome domains will be handled with domain-specific extraction fields and appraisal tools, synthesized separately, and then integrated into a final narrative that examines coherence and trade-offs across domains.

## Methods

### Protocol and Registration

The systematic review will adhere to the PRISMA-P 2015 statement [30] and will be reported in accordance with the PRISMA 2020 guidelines [31]. The protocol is registered with the International Prospective Register of Systematic Reviews (PROSPERO; registration number CRD420261351022). Any amendments to this protocol will be documented, dated, and reported transparently in the final review manuscript.

### Eligibility Criteria

Eligibility criteria are defined using the PICOS (population, intervention, comparator, outcomes, study design) framework [32] and are summarized in Table 1. The review focuses on primary bariatric or metabolic procedures as the index intervention. Revisional or conversion procedures are not eligible as the index intervention; studies reporting mixed primary and revisional cohorts will be included only where outcomes for primary procedures are separately extractable and will otherwise be excluded. Clinical and economic outcomes are considered primary, whereas system-level resource utilization outcomes are designated as exploratory; eligible studies must report at least one outcome of interest. For the exploratory domain, length of stay is defined as the index-admission duration from operation (or admission) to discharge, as reported by study authors; preoperative pathway duration is defined as the interval from surgical qualification or referral to surgery; outpatient care burden is defined as the number and frequency of preoperative and postoperative outpatient visits; and readmission is defined using author-defined 30-day, 90-day, or other reported windows. Because these definitions vary by country, insurance system, and referral model, author-reported definitions and the health care system context will be recorded for each study to support contextual interpretation.

**Table 1.** Eligibility criteria (PICOS^a^ framework).

Parameter	Inclusion criteria	Exclusion criteria
Population	Adults aged ≥18 years with obesity (BMI≥30 kg/m^2^) Obesity diagnosed clinically or via reported BMI Patients undergoing primary bariatric surgery	Mixed-age populations Adolescent or pediatric populations (<18 y) Endoscopic or pharmacological interventions without a surgical component
Intervention	Any primary bariatric or metabolic procedure, including RYGB^b^, SG^c^, OAGB^d^, LAGB^e^, BPD±DS^f^, and other established bariatric procedures	Intragastric balloon placement Endoscopic sleeve gastroplasty Pharmacotherapy alone Nonsurgical weight-reduction programs without a surgical component Revisional/conversion procedures as the index intervention
Comparator	Another bariatric procedure (eg, RYGB vs OAGB) Nonsurgical standard or usual care No comparator (single-arm studies eligible)	—^g^
Outcomes	At least one clinical, economic, or system-level outcome of interest (see Data Extraction section)	No relevant outcome reported
Study design	RCTs^h^ Prospective and retrospective cohort studies Case-control studies Cross-sectional studies with relevant outcome data Economic evaluation studies with extractable primary data Resource utilization studies	Case reports or series with <10 participants Qualitative studies Reviews and meta-analyses Editorials, letters, commentaries, and conference abstracts without full text Economic models and health technology assessments requiring reconstruction of inputs without extractable empirical data Gray literature
Other	Any health care setting; no geographical restriction English-language publications Published between January 1, 2020, and December 31, 2025 Full-text peer-reviewed journal articles	Non–English-language publications Publication date outside 2020‐2025

a PICOS: population, intervention, comparator, outcomes, study design.

b RYGB: Roux-en-Y gastric bypass.

c SG: sleeve gastrectomy.

d OAGB: one-anastomosis gastric bypass.

e LAGB: laparoscopic adjustable gastric banding.

f BPD±DS: biliopancreatic diversion with or without duodenal switch.

g Not applicable.

h RCT: randomized controlled trial.

### Information Sources

Four electronic bibliographic databases will be searched: MEDLINE (via PubMed), Embase, Scopus, and Web of Science. Embase has been included to improve retrieval of European and economic literature indexed with Emtree terms that may not be fully captured by the other databases. Cochrane CENTRAL was considered but not prioritized because the review deliberately includes a broad range of nonrandomized, observational, and economic study designs that extend well beyond the controlled-trial focus of CENTRAL. EconLit and the (now archived) National Health Service (NHS) Economic Evaluation Database (EED) were considered but not searched owing to access constraints and the archival status of NHS EED. Clinical trial registries were not searched because the predominant eligible designs (cohort, case-control, and economic evaluations) are not routinely registered. These decisions are acknowledged in the *Limitations* section.

### Search Strategy

A systematic search strategy will be developed and implemented in accordance with PRISMA-P guidance [30] and the PRESS (Peer Review of Electronic Search Strategies) recommendations [33]. A dual-track design reflects the distinct information needs of the 2 secondary outcome domains and maximizes sensitivity without inflating false-positive rates through inappropriate block combinations. The economic track applies a 4-block AND structure combining block 1 (population), block 2 (bariatric procedures), block 3a (economic terms), and block 4 (clinical outcome terms). The system-level track applies a 3-block AND structure combining block 1 (population), block 2 (bariatric procedures), and block 3b (system-level resource utilization terms). Block 1 covers the index condition (obesity or overweight); block 2 covers all eligible primary bariatric procedures; block 3a covers direct costs, economic evaluations, and cost-effectiveness measures (cost, costs, health care costs, hospital costs, economic evaluation, cost-effectiveness, cost-utility, cost-benefit, ICER, QALY, quality-adjusted life year, net monetary benefit, budget impact, and willingness to pay); block 3b covers hospital throughput, care pathways, and resource-use metrics (length of stay, LOS, hospital stay, readmission, outpatient visits, emergency visits, resource utilization, waiting time, waiting list, preoperative pathway, throughput, bed occupancy, operative volume, enhanced recovery, enhanced recovery after surgery); and block 4 covers weight loss, metabolic outcomes, complications, and mortality (weight loss, body weight change, BMI change, total weight loss, excess weight loss, TWL, excess weight loss (EWL), remission, improvement, complications, reoperations, and mortality). Each strategy is tailored to the indexing, controlled vocabulary, and syntax of the respective database. The full database-specific strategies are provided in Textbox 1.

Textbox 1. **Database-specific strategies.**

**Scopus—Track 1 (Economic)**

- TITLE-ABS-KEY (obes* OR overweight*) AND TITLE-ABS-KEY (“bariatric surgery” OR “weight loss surgery” OR “gastric bypass” OR “Roux-en-Y gastric bypass” OR “sleeve gastrectomy” OR “one-anastomosis gastric bypass” OR “mini-gastric bypass” OR “gastric band*” OR “vertical banded gastroplasty” OR “biliopancreatic diversion” OR “biliopancreatic diversion with duodenal switch” OR “duodenal switch”) AND TITLE-ABS-KEY (cost OR costs OR “healthcare cost*” OR “hospital cost*” OR “economic evaluation*” OR “cost-effectiveness” OR “cost-utility” OR “cost-benefit” OR ICER OR QALY OR QALYs OR “quality-adjusted life year*” OR “net monetary benefit” OR NMB OR “budget impact” OR “willingness to pay”) AND TITLE-ABS-KEY (“weight loss” OR “body weight change” OR “BMI change” OR “total weight loss” OR “excess weight loss” OR TWL OR EWL OR remission OR improvement OR complication* OR reoperation* OR mortality) AND PUBYEAR>2019 AND PUBYEAR<2026 AND LIMIT-TO (LANGUAGE, “English”)

**Scopus—Track 2 (System-Level)**

- TITLE-ABS-KEY (obes* OR overweight*) AND TITLE-ABS-KEY (“bariatric surgery” OR “weight loss surgery” OR “gastric bypass” OR “Roux-en-Y gastric bypass” OR “sleeve gastrectomy” OR “one-anastomosis gastric bypass” OR “mini-gastric bypass” OR “gastric band*” OR “vertical banded gastroplasty” OR “biliopancreatic diversion” OR “biliopancreatic diversion with duodenal switch” OR “duodenal switch”) AND TITLE-ABS-KEY (“length of stay” OR LOS OR “hospital stay” OR readmission* OR “hospital readmission” OR “outpatient visit*” OR “emergency visit*” OR “resource utili*” OR “waiting time” OR “waiting list” OR “preoperative pathway” OR “throughput” OR “bed occupancy” OR “operative volume” OR “enhanced recovery” OR ERAS) AND PUBYEAR>2019 AND PUBYEAR<2026 AND LIMIT-TO (LANGUAGE, “English”)

**MEDLINE/PubMed—Track 1 (Economic)**

- (“Obesity”[Mesh] OR obes*[tiab] OR overweight*[tiab]) AND (“Bariatric Surgery”[Mesh] OR “bariatric surgery”[tiab] OR “weight loss surgery”[tiab] OR “gastric bypass”[tiab] OR “Roux-en-Y gastric bypass”[tiab] OR “sleeve gastrectomy”[tiab] OR “one-anastomosis gastric bypass”[tiab] OR “mini-gastric bypass”[tiab] OR “adjustable gastric banding”[tiab] OR “gastric band*“[tiab] OR “vertical banded gastroplasty”[tiab] OR “biliopancreatic diversion”[tiab] OR “biliopancreatic diversion with duodenal switch”[tiab] OR “duodenal switch”[tiab]) AND (“Costs and Cost Analysis”[Mesh] OR cost[tiab] OR costs[tiab] OR “healthcare cost*“[tiab] OR “hospital cost*“[tiab] OR “economic evaluation*“[tiab] OR “cost-effectiveness”[tiab] OR “cost-utility”[tiab] OR “cost-benefit”[tiab] OR ICER[tiab] OR QALY[tiab] OR QALYs[tiab] OR “quality-adjusted life year*“[tiab] OR “net monetary benefit”[tiab] OR NMB[tiab] OR “budget impact”[tiab] OR “willingness to pay”[tiab]) AND (“weight loss”[tiab] OR “body weight change”[tiab] OR “BMI change”[tiab] OR “total weight loss”[tiab] OR “excess weight loss”[tiab] OR TWL[tiab] OR EWL[tiab] OR remission[tiab] OR improvement[tiab] OR complication*[tiab] OR reoperation*[tiab] OR mortality[tiab]) AND (“2020/01/01”[PDAT] : “2025/12/31”[PDAT]) AND (english[lang])

**MEDLINE/PubMed—Track 2 (System-Level)**

- (“Obesity”[Mesh] OR obes*[tiab] OR overweight*[tiab]) AND (“Bariatric Surgery”[Mesh] OR “bariatric surgery”[tiab] OR “weight loss surgery”[tiab] OR “gastric bypass”[tiab] OR “Roux-en-Y gastric bypass”[tiab] OR “sleeve gastrectomy”[tiab] OR “one-anastomosis gastric bypass”[tiab] OR “mini-gastric bypass”[tiab] OR “adjustable gastric banding”[tiab] OR “gastric band*“[tiab] OR “vertical banded gastroplasty”[tiab] OR “biliopancreatic diversion”[tiab] OR “biliopancreatic diversion with duodenal switch”[tiab] OR “duodenal switch”[tiab]) AND (“Length of Stay”[Mesh] OR “Patient Readmission”[Mesh] OR “length of stay”[tiab] OR LOS[tiab] OR “hospital stay”[tiab] OR readmission*[tiab] OR “hospital readmission”[tiab] OR “outpatient visit*“[tiab] OR “emergency visit*“[tiab] OR “resource utili*“[tiab] OR “waiting time”[tiab] OR “waiting list”[tiab] OR “preoperative pathway”[tiab] OR “throughput”[tiab] OR “bed occupancy”[tiab] OR “operative volume”[tiab] OR “enhanced recovery”[tiab] OR ERAS[tiab]) AND (“2020/01/01”[PDAT]: “2025/12/31”[PDAT]) AND (english[lang])

**EMBASE—Track 1 (Economic)**

- (“obesity”/exp OR obes*:ab,ti OR overweight*:ab,ti) AND (“bariatric surgery”/exp OR “bariatric surgery”:ab,ti OR “weight loss surgery”:ab,ti OR “gastric bypass”:ab,ti OR “roux-en-y gastric bypass”:ab,ti OR “sleeve gastrectomy”:ab,ti OR “one-anastomosis gastric bypass”:ab,ti OR “mini-gastric bypass”:ab,ti OR “gastric banding”:ab,ti OR “vertical banded gastroplasty”:ab,ti OR “biliopancreatic diversion”:ab,ti OR “duodenal switch”:ab,ti) AND (“health care cost”/exp OR cost:ab,ti OR costs:ab,ti OR “healthcare cost*”:ab,ti OR “hospital cost*”:ab,ti OR “economic evaluation*”:ab,ti OR “cost effectiveness”:ab,ti OR “cost utility”:ab,ti OR “cost benefit”:ab,ti OR ICER:ab,ti OR QALY:ab,ti OR QALYs:ab,ti OR “quality adjusted life year*”:ab,ti OR “net monetary benefit”:ab,ti OR NMB:ab,ti OR “budget impact”:ab,ti OR “willingness to pay”:ab,ti) AND (“weight loss”:ab,ti OR “body weight change”:ab,ti OR “BMI change”:ab,ti OR “total weight loss”:ab,ti OR “excess weight loss”:ab,ti OR TWL:ab,ti OR EWL:ab,ti OR remission:ab,ti OR improvement:ab,ti OR complication*:ab,ti OR reoperation*:ab,ti OR mortality:ab,ti) AND [english]/lim AND [2020‐2025]/py

**EMBASE—Track 2 (System-Level)**

- (“obesity”/exp OR obes*:ab,ti OR overweight*:ab,ti) AND (“bariatric surgery”/exp OR “bariatric surgery”:ab,ti OR “weight loss surgery”:ab,ti OR “gastric bypass”:ab,ti OR “roux-en-y gastric bypass”:ab,ti OR “sleeve gastrectomy”:ab,ti OR “one-anastomosis gastric bypass”:ab,ti OR “mini-gastric bypass”:ab,ti OR “gastric banding”:ab,ti OR “vertical banded gastroplasty”:ab,ti OR “biliopancreatic diversion”:ab,ti OR “duodenal switch”:ab,ti) AND (“length of stay”/exp OR “length of stay”:ab,ti OR LOS:ab,ti OR “hospital stay”:ab,ti OR readmission*:ab,ti OR “hospital readmission”/exp OR “outpatient visit*”:ab,ti OR “emergency visit*”:ab,ti OR “resource utili*”:ab,ti OR “waiting time”:ab,ti OR “waiting list”:ab,ti OR “preoperative pathway”:ab,ti OR throughput:ab,ti OR “bed occupancy”:ab,ti OR “operative volume”:ab,ti OR “enhanced recovery”:ab,ti OR ERAS:ab,ti) AND [english]/lim AND [2020‐2025]/py

**Web of Science—Track 1 (Economic)**

- TS=(obes* OR overweight*) AND TS=(“bariatric surgery” OR “weight loss surgery” OR “gastric bypass” OR “Roux-en-Y gastric bypass” OR “sleeve gastrectomy” OR “one-anastomosis gastric bypass” OR “mini-gastric bypass” OR “gastric band” OR “gastric banding” OR “gastric banded” OR “vertical banded gastroplasty” OR “biliopancreatic diversion” OR “duodenal switch”) AND TS=(cost OR costs OR (healthcare NEAR/0 cost*) OR (hospital NEAR/0 cost*) OR (economic NEAR/0 evaluat*) OR “cost-effectiveness” OR “cost-utility” OR “cost-benefit” OR ICER OR QALY OR QALYs OR “quality-adjusted life year*” OR “net monetary benefit” OR NMB OR “budget impact” OR “willingness to pay”) AND TS=(“weight loss” OR “body weight change” OR “BMI change” OR “total weight loss” OR “excess weight loss” OR TWL OR EWL OR remission OR improvement OR complication* OR reoperation* OR mortality) AND PY=(2020‐2025) AND LA=(English)

**Web of Science—Track 2 (System-Level)**

- TS=(obes* OR overweight*) AND TS=(“bariatric surgery” OR “weight loss surgery” OR “gastric bypass” OR “Roux-en-Y gastric bypass” OR “sleeve gastrectomy” OR “one-anastomosis gastric bypass” OR “mini-gastric bypass” OR “gastric band” OR “gastric banding” OR “gastric banded” OR “vertical banded gastroplasty” OR “biliopancreatic diversion” OR “duodenal switch”) AND TS=(“length of stay” OR LOS OR “hospital stay” OR readmission* OR “hospital readmission” OR (outpatient NEAR/0 visit*) OR (emergency NEAR/0 visit*) OR (resource NEAR/0 utili*) OR “waiting time” OR “waiting list” OR “preoperative pathway” OR “throughput” OR “bed occupancy” OR “operative volume” OR “enhanced recovery” OR ERAS) AND PY=(2020‐2025) AND LA=(English)

### Study Selection

Study selection will follow a 2-stage process in accordance with PRISMA 2020 standards. Search results from all databases will be imported into Zotero, and duplicate records will be identified and removed using an automatic tool with manual verification. At stage 1, titles and abstracts will be screened against the eligibility criteria and classified as included (proceed to full-text screening) or excluded; records whose eligibility is uncertain from the title and abstract alone will proceed to full-text screening. At stage 2, full texts will be obtained for all potentially eligible records, and reasons for exclusion will be documented using predefined categories (ineligible population, intervention, comparator, or design; no relevant outcomes; publication date outside 2020‐2025; non-English language; conference abstract, letter, or editorial; full text unavailable; or other).

Screening will be performed by a single primary reviewer (MB), with an independent second reviewer duplicating at least 20% of records at both the title-and-abstract and the full-text stages. This verification sample will be allocated deliberately rather than drawn at random. A calibration tranche comprising the first 100 titles and abstracts and the first 20 full-text reports will be screened in duplicate before proper screening begins; disagreements arising will be examined jointly, and the eligibility criteria clarified before the remaining records are screened. The balance of the verification sample will be concentrated on full-text reports, studies progressing to inclusion, and records flagged as uncertain by the primary reviewer, since these are the decisions whose consequences for the review cannot be recovered at a later stage. Disagreements will be resolved by discussion, and a third senior reviewer will adjudicate where consensus cannot be reached. The number of records duplicated, the disagreements arising and their resolution, and the verification fraction achieved will be reported for each stage in the completed review. The single-primary-reviewer design reflects the constraints of a doctoral research project, in which the candidate is required to conduct the core work independently, and no funded review team is available, and is acknowledged as a limitation.

### Data Extraction

A standardized data extraction form will be developed in spreadsheet software and piloted on a sample of 5 included studies to assess clarity, completeness, and consistency before being refined as necessary. Extraction will be performed primarily by a single reviewer using predefined variables and operational definitions, and, consistent with the screening approach, at least 20% of included studies will be independently re-extracted by a second reviewer, with priority given to the values that enter the synthesis, namely the priority clinical outcomes and all extracted cost estimates, with discrepancies resolved through consensus. Where data are missing or unclear, corresponding authors of the original studies will be contacted by email (up to 2 attempts over 4 weeks). The categories of information to be extracted are summarized in Table 2. For the 3 outcome domains, distinct extraction fields are used so that clinical, economic, and system-level data can be appraised and synthesized separately before integration.

**Table 2.** Data extraction variables by domain.

Domain	Variables extracted
Study characteristics	First author and publication year; study design; country and health care setting; study period (enrollment and data collection dates)
Population	Sample size (total and per group); age (mean/median/range); sex distribution; baseline BMI and weight; comorbidity profile (type 2 diabetes, hypertension, dyslipidemia, OSA^a^); previous weight-loss interventions; inclusion or exclusion criteria
Intervention	Procedure type (RYGB^b^; OAGB^c^; LAGB^d^; BPD±DS^e^; other); surgical approach (laparoscopic, open, robotic); use of ERAS^f^
Comparator	Type of comparator (another bariatric procedure, nonsurgical care, usual care, none) and comparator details
Follow-up	Duration of follow-up; follow-up time points (eg, 6, 12, 24, 36, 60 mo); loss to follow-up rate
Clinical outcomes	%TWL^g^ as the primary metric and durability over time; remission or improvement of type 2 diabetes, hypertension, dyslipidemia, and OSA; overall and severity-classified complications (Clavien-Dindo); specific complications; reoperation rate; 30-day and long-term mortality; author-defined clinical success or failure
Economic outcomes	Direct costs (hospitalization, surgery, complications, readmissions, outpatient, pharmacotherapy, diagnostics); ICER^h^; cost per unit of clinical outcome; QALYs^i^ and cost per QALY; incremental cost-utility ratio; NMB^j^; budget impact and cost-benefit results; original currency, price year, and analytic perspective; where the price year is not stated by the authors, the level of the prespecified hierarchy is used to establish it
System-level outcomes	Length of stay; readmission rates (30- or 90-day or other); preoperative pathway length; number/frequency of outpatient visits; use of ERAS; health care–system context (eg, NHS^k^, insurance-based, mixed) and author-applied definitions and time points

a OSA: obstructive sleep apnea.

b RYGB: Roux-en-Y gastric bypass.

c OAGB: one-anastomosis gastric bypass.

d LAGB: laparoscopic adjustable gastric banding.

e BPD±DS: biliopancreatic diversion with or without duodenal switch.

f ERAS: enhanced recovery after surgery.

g %TWL: percentage total weight loss.

h ICER: incremental cost-effectiveness ratio.

i QALY: quality-adjusted life year.

j NMB: net monetary benefit.

k NHS: National Health Service.

### Risk of Bias and Quality Assessment

Risk of bias (RoB) will be assessed by a single reviewer using tools appropriate to each study design, with at least 20% of assessments independently validated by a second reviewer, with priority given to studies rated at high or critical RoB, where the judgment determines the sensitivity analysis and the GRADE rating, and with discrepancies resolved by discussion (third-reviewer adjudication where required). For randomized controlled trials (RCTs), the Cochrane RoB 2 tool will be used, with each domain rated as low risk, some concerns, or high risk, and an overall judgment per outcome [34]. For nonrandomized interventional and observational studies, ROBINS-I (Risk of Bias in Nonrandomized Studies of Interventions) will be used, with domains rated as low, moderate, serious, or critical risk, or no information, and an overall judgment per study [35]. For economic evaluations, the completeness of reporting will be assessed with the CHEERS (Consolidated Health Economic Evaluation Reporting Standards) 2022 checklist [36]; because CHEERS evaluates reporting quality rather than methodological validity or RoB, the methodological quality of economic evaluations will additionally be appraised using the Consensus on Health Economic Criteria (CHEC) checklist [37]. CHEERS findings will therefore be interpreted as indicators of reporting transparency, while CHEC findings will inform judgments of internal validity. The tools applied to each design are summarized in Table 3.

**Table 3.** Critical appraisal tools by study design.

Study design	Tool	Ratings/purpose
Randomized controlled trials	Cochrane RoB^a^ 2	Low/some concerns/high risk
Nonrandomized and observational studies	ROBINS-I^b^	Low/moderate/serious/critical/no information
Economic evaluations (reporting)	CHEERS^c^ 2022	Completeness of reporting
Economic evaluations (methodological quality)	CHEC^d^ checklist	Internal/methodological validity

a RoB: risk of bias.

b ROBINS-I: Risk of Bias in Nonrandomized Studies of Interventions.

c CHEERS: Consolidated Health Economic Evaluation Reporting Standards.

d CHEC: Consensus on Health Economic Criteria.

RoB and quality judgments will be integrated into the synthesis and certainty rating, rather than being used only descriptively. Studies rated at high or critical RoB (RoB 2 or ROBINS-I) or at low methodological quality (CHEC) will be flagged, and their influence on conclusions will be explored through narrative sensitivity analysis that examines whether the direction or consistency of findings changes when lower-quality studies are set aside. These judgments will directly inform the RoB domain of the GRADE assessment [38] and may downgrade the certainty of evidence for the affected outcomes. RoB assessments will be summarized in tables for each included study and accompanied by a narrative description across the evidence base.

### Data Synthesis

The primary method of integration will be a narrative synthesis, conducted in accordance with established guidance on the conduct of narrative synthesis [39] and reported in line with the Synthesis Without Meta-Analysis (SWiM) reporting guideline [40]. This approach is justified by the anticipated heterogeneity in study designs, outcome definitions, measurement methods, follow-up durations, and health care settings, as confirmed by preliminary scoping that identified considerable variation in clinical outcome definitions (weight-loss metrics, remission criteria, complication classifications), economic outcomes (cost components, analytic perspectives, valuation methods, currency and price year, discounting), and system-level outcomes (readmission criteria and resource utilization metrics). The narrative synthesis will tabulate study characteristics, populations, interventions and comparators, and outcomes, grouped by outcome domain and design; will explore relationships by examining variation by procedure type, geographical region, and study quality; and will assess robustness by integrating RoB judgments into the interpretation. Results will be presented in a structured narrative supported by evidence tables, and findings from the clinical, economic, and system-level domains will be integrated in a concluding narrative that examines coherence and trade-offs across domains.

To make synthesis decisions transparent, priority outcomes and comparison parameters are prespecified. The priority clinical outcomes are %TWL at 12 and 24 months, remission of type 2 diabetes, and overall and severe (Clavien-Dindo≥III) complications; the priority economic outcome is cost per QALY gained. Prespecified subgroup categories are procedure type (RYGB, SG, OAGB, and others), follow-up window (≤12, 13‐36, and >36 mo), and health care setting or geographical region. Before any quantitative pooling is considered, a minimum comparability standard must be met: studies must address the same priority outcome, at a comparable time point or window, using comparable outcome definitions, in a comparable population and procedure category.

Quantitative synthesis is not planned a priori. A meta-analysis will be considered only if, during extraction and synthesis, a sufficiently homogeneous subset of studies is identified and organizational feasibility allows it. The minimum requirements are that at least 3 studies report the same priority outcome using comparable definitions, measurement methods, and time points; that there is clinical and methodological justification for pooling and that the comparability standard described above is satisfied; that effect estimates and variance are adequately reported or derivable; and that sufficient expertise and resources are available. For clinically heterogeneous bariatric outcomes spanning different procedures, follow-up periods, and settings, the 3-study figure is treated as a necessary but not sufficient condition, and a larger number of comparable studies will generally be required before pooling is judged appropriate; the final decision will rest on documented clinical and methodological judgment rather than the study count alone. Where meta-analysis is performed, random-effects models will be applied, heterogeneity will be quantified using *I*^2^ and τ^2^ statistics [41], and the results will be presented in forest plots with appropriate caveats. Subgroup or sensitivity analyses will be undertaken only where justified by the data, and all pooling decisions will be documented with explicit justification.

Cost data will be extracted in the original currency and price year as reported by the study authors. Harmonization will follow established guidance on adjusting for inflation and currency differences within health economic studies [42] and will apply the 2-stage computation implemented in the CCEMG-EPPI-Centre Cost Converter (Campbell and Cochrane Economics Methods Group–Evidence for Policy and Practice Information and Co-ordinating Centre) [43]. Costs will first be inflated within the original currency from the reported price year to a common target price year using country-specific gross domestic product deflators, and the inflated values will then be converted to a common currency using purchasing power parity conversion factors for that same year. Gross domestic product deflators are preferred to health-specific consumer price indices because the latter reflect prices faced by consumers out of pocket rather than the cost of providing care and are therefore poorly suited to countries with predominantly publicly financed health care. Purchasing power parities are preferred over market exchange rates because they eliminate differences in price levels between countries, and inflating before converting avoids the distortion that arises when the 2 operations are applied in the reverse order.

Where the price year is not stated, it will be established by a prespecified hierarchy: the year of the unit-cost source cited by the authors (eg, a national tariff, reference-cost schedule, or reimbursement catalog); failing that, the final year of the period over which resource-use data were collected; failing that, for modeling studies, the publication year of the principal cost data source used in the model; and failing all of these, the year of publication minus one. The level of the hierarchy applied will be recorded for each study and reported in the evidence table.

The target price year will be 2024, and costs will be expressed in euros, with Germany specified as the target country. Gross domestic product deflators will be obtained from the International Monetary Fund World Economic Outlook database (April 2026 edition) and purchasing power parity conversion factors from the OECD purchasing power parities for gross domestic product dataset. Values for 2024 are observed rather than projected for all countries covered by these datasets, which is the reason for selecting this target year. Because the conversion is applied to a large number of estimates, it will be executed by a purpose-written script implementing the computation described above, rather than being entered manually. A random sample of conversions will be reproduced independently in the CCEMG-EPPI-Centre Cost Converter to confirm that the computation is correctly implemented. Exact agreement is not expected, because the current version of that tool draws on earlier editions of the same source datasets; residual differences attributable to dataset revision will be quantified and reported. The edition and date of access of each source dataset will be stated in the completed review, since both are periodically revised.

If more than one-quarter of the included economic studies were conducted in countries not covered by the OECD purchasing power parity dataset, or if excluding such studies would remove an entire continent from the harmonized comparison, the conversion will instead be performed for all studies using the implied purchasing power parity series from the World Economic Outlook database. Conversion factors from the 2 sources will not be combined within a single comparison, and the decision, together with the figures on which it was based, will be reported.

One sensitivity analysis is prespecified. It repeats the conversion using whichever of the 2 purchasing power parity series was not used for the main analysis in order to establish whether cross-country comparisons are robust to the choice of conversion source. Harmonization will be applied to prespecified summary quantities rather than to every reported cost item, namely total cost per patient by procedure and comparator, incremental costs, and ICERs; individual cost components will be retained in the original currency and price year. ICERs will additionally be reported in the original currency alongside the cost-effectiveness threshold applied in the source study, because a converted ratio is not interpretable against a threshold from another jurisdiction. Because harmonization to a common currency and price year does not remove differences in analytic perspective, time horizon, or discount rate, these attributes will be extracted for every economic estimate; harmonized cost comparisons will be presented within rather than across perspective and time-horizon strata, and comparisons between studies will rely primarily on within-study relative measures, with harmonized absolute values used descriptively. The original currency, reported price year, target price year, inflation index, and conversion factors applied will be reported transparently in the evidence table for each study; where harmonization is not feasible owing to insufficient reporting of currency or cost components, or where no basis for establishing the price year can be identified, the affected studies will be retained in the narrative synthesis but excluded from tabular cost comparisons, with the reason documented.

Two forms of reporting bias will be assessed separately. Publication bias refers to the selective nonpublication of entire studies based on the direction or magnitude of their results; because the primary synthesis is narrative, formal statistical methods such as funnel plots or the Egger test will be applied only if a meta-analysis is performed and includes at least 10 studies per outcome [44], and, in their absence, publication bias will be evaluated qualitatively by examining gaps or imbalances across outcome domains and the plausibility of the observed distribution of findings. Outcome reporting bias refers to the selective reporting of a subset of measured outcomes within a published study; for RCTs, this will be assessed within the RoB 2 evaluation, and for nonrandomized studies within ROBINS-I, and, where protocols or trial registrations are available, reported outcomes will be compared with prespecified outcomes to identify discrepancies. The potential influence of both forms of bias will be addressed in the *Discussion* section.

### Certainty of Evidence

The overall certainty of the cumulative evidence for each priority outcome will be assessed using the GRADE approach and rated across the domains of RoB, inconsistency, indirectness, imprecision, and publication bias, with certainty classified as high, moderate, low, or very low. Summary-of-findings tables will be produced for the priority outcomes. For economic outcomes, certainty will be informed jointly by the CHEERS 2022 (reporting) and CHEC (methodological quality) appraisals.

### Ethical Considerations

Ethical approval is not required for this systematic review, as it uses only previously published data and does not involve the direct participation of human participants. Individual-level patient data will not be collected, and no identifiable personal information will be accessed.

### Dissemination Plan

The results of the completed systematic review will be submitted to a peer-reviewed journal focusing on bariatric surgery, health outcomes, health economics, or public health, and may be presented at relevant scientific meetings. The PROSPERO registration number will be reported in the final review, and any protocol amendments will be documented and dated.

## Results

This protocol describes a systematic review that is currently in progress; no findings are yet available. The protocol was registered with PROSPERO (CRD420261351022) on April 15, 2026. Database searches across MEDLINE/PubMed, Embase, Scopus, and Web of Science were conducted on August 1, 2026, and identified 9400 records, of which 3868 remained after duplicate removal. Title-and-abstract screening is scheduled to begin on August 15, 2026. Full-text screening, data extraction, and RoB assessment are expected to be completed by the end of 2026, and the results of the completed review are anticipated for publication in the first half of 2027.

The flow of records through identification, screening, eligibility assessment, and inclusion will be reported using a PRISMA 2020 flow diagram. The diagram template is shown in Figure 1. Final record counts and exclusion reasons will be reported once the searches and screening are finalized and will appear in the completed review.

**Figure 1.**
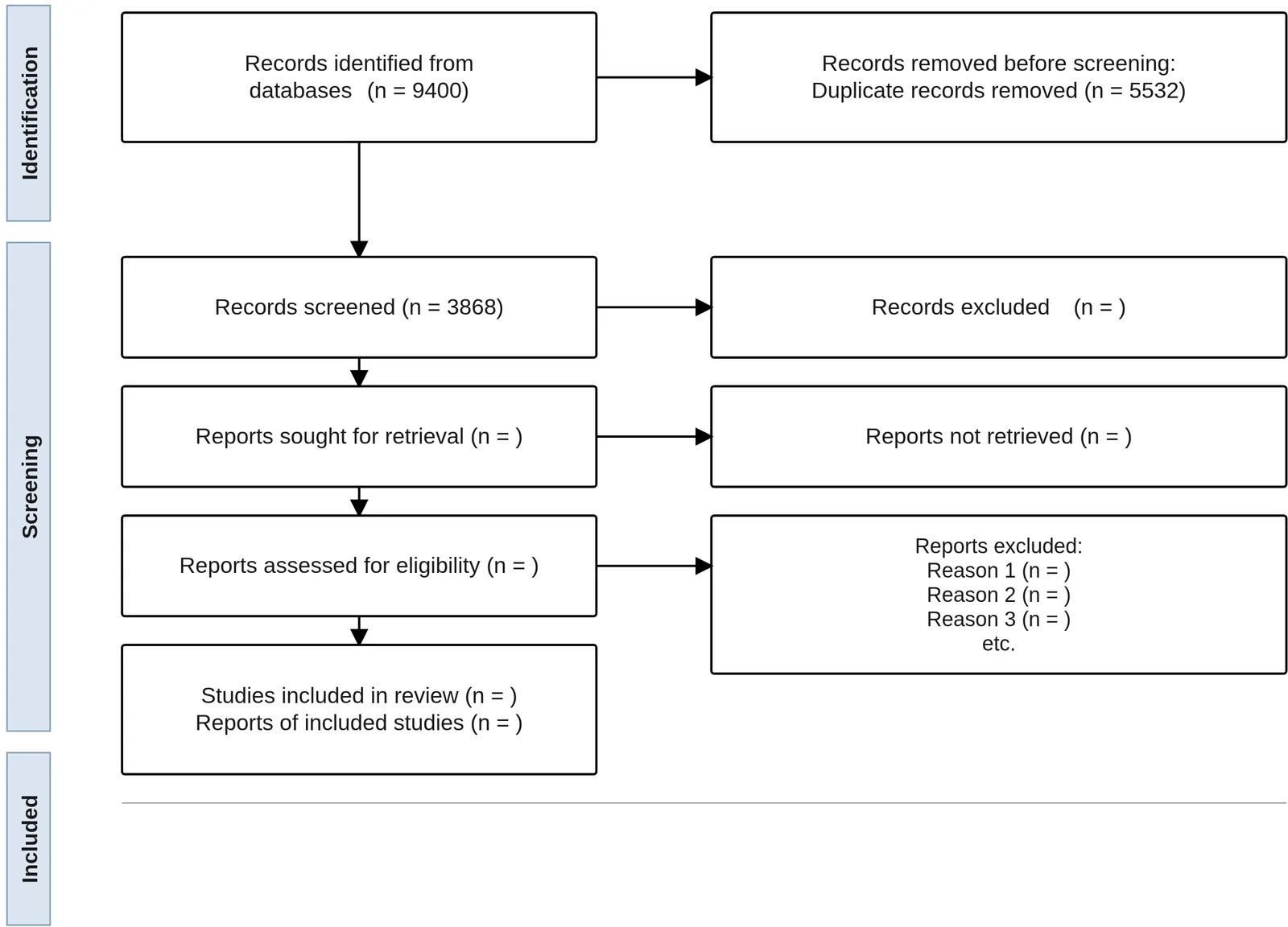
Preferred Reporting Items for Systematic Reviews and Meta-Analysis (PRISMA) 2020 flow diagram (template).

## Discussion

### Principal Findings

This protocol sets out a transparent and reproducible methodology for an integrated synthesis of the clinical, economic, and system-level outcomes of bariatric surgery in adults. We anticipate that the completed review will characterize whether that contemporary (2020‐2025) evidence continues to support substantial and durable weight loss and meaningful rates of metabolic remission following primary bariatric procedures, while also clarifying how these clinical benefits relate to direct costs, cost-effectiveness, and the demands that bariatric programs place on health care delivery. By appraising and synthesizing the 3 domains separately and then integrating them, the review is designed to surface trade-offs—for example, between procedure-specific clinical effectiveness and the resource intensity of perioperative pathways—that single-domain reviews cannot capture. Because the synthesis is primarily narrative, the principal contribution is expected to be an organized, critically appraised map of contemporary evidence and its gaps rather than pooled effect estimates.

### Comparison With Prior Work

Most existing systematic reviews of bariatric surgery outcomes draw on data collected before 2020 and tend to address clinical effectiveness or cost-effectiveness in isolation. By restricting inclusion to studies published between 2020 and 2025, and by deliberately spanning clinical, economic, and system-level domains, this review is intended to update and extend that prior work in a period marked by an evolving procedure mix (notably the continued growth of SG and OAGB) [22], changing patient case mix, and shifting health care financing. The explicit harmonization of cost data to a common price year and currency, and the separation of reporting-quality (CHEERS) from methodological-quality (CHEC) appraisal of economic evaluations, are intended to address recognized inconsistencies in how economic evidence has previously been compared across settings.

### Strengths and Limitations

The principal strengths of this protocol are its prospective registration, adherence to PRISMA-P and PRISMA 2020 standards, a dual-track search across 4 major databases, including Embase, prespecified priority outcomes and comparability criteria that guard against an overly broad descriptive review, and a structured plan for integrating RoB judgments into GRADE. Several limitations should be acknowledged. First, screening, extraction, and RoB assessment are performed primarily by a single reviewer, reflecting the resource constraints of a doctoral project; this is mitigated, but not eliminated, by independent duplication of at least 20% of records focused on full texts and included studies, with interreviewer agreement reported. Second, restricting inclusion to English-language publications may introduce language bias and exclude relevant non-English studies; this restriction is adopted to support accurate interpretation of complex clinical and economic data within existing resources and is acknowledged as a potential source of bias. Third, the anticipated clinical, methodological, and contextual heterogeneity may preclude quantitative pooling, limiting the ability to provide aggregate effect estimates; narrative synthesis is nonetheless preferred to avoid misleading conclusions from inappropriate pooling. Fourth, economic outcomes are highly context-dependent, shaped by health care system structure, reimbursement models, and valuation methods, so findings from one setting may not generalize to others. Finally, clinical trial registries were not searched because the predominant eligible designs are not routinely registered, which may contribute to publication bias; this is addressed qualitatively. In addition, the system-level domain is exploratory, and its indicators are defined inconsistently across health care systems, so findings in this domain will support description and hypothesis generation rather than firm conclusions. Cost harmonization also relies on national price indices and purchasing power parities that are themselves estimates, and harmonized figures should therefore be read as approximate rather than exact equivalents. In particular, conversion factors are based on purchasing power parities for gross domestic product rather than health-specific purchasing power parities, which are not available for all countries and price years likely to be represented; since relative price structures in the health sector differ from those in the wider economy, this substitution introduces further imprecision into cross-country cost comparisons. Under the OECD series, conversion factors are available only for member countries, so studies conducted elsewhere are excluded from harmonized cost comparisons unless the substitution described in the *Methods* section is triggered. Harmonized values also depend on the edition of the source datasets used, since purchasing power parities are revised following international benchmark exercises. Such revisions are substantial for the International Monetary Fund series and marginal for the OECD series, which is why the OECD series is used by default and the International Monetary Fund series only when country coverage makes it necessary.

### Future Directions

By mapping where contemporary evidence is concentrated and where it is sparse, the review is intended to identify priorities for future research—for example, standardized reporting of %TWL and complication severity, prospective economic evaluations conducted alongside clinical studies, and consistent definitions of system-level indicators, such as preoperative pathway duration. Such standardization would make future evidence more amenable to quantitative synthesis and more directly useful for cross-country comparison and capacity planning.

### Conclusions

This protocol presents a transparent and comprehensive methodology for a systematic review of the most recent evidence on the clinical, economic, and system-level outcomes of bariatric surgery in adults with obesity. By synthesizing studies published between 2020 and 2025 across multiple outcome domains and study designs, the review aims to produce an up-to-date, critically appraised evidence base to inform clinicians, health care administrators, policymakers, and health economists, and to guide future research directions. The conclusions of the completed review will be drawn only to the extent supported by the identified evidence and its assessed certainty.

## Supplementary material

10.2196/98976 Checklist 1 PRISMA-P checklist.

## Abbreviations

%TWL: percentage total weight loss
BPD: biliopancreatic diversion
CCEMG-EPPI-Centre: Campbell and Cochrane Economics Methods Group Evidence for Policy and Practice Information and Co-ordinating Centre
CHEC: Consensus on Health Economic Criteria
CHEERS: Consolidated Health Economic Evaluation Reporting Standards
DS: duodenal switch
EED: Economic Evaluation Database
EWL: excess weight loss
GRADE: Grading of Recommendations, Assessment, Development and Evaluations
ICER: incremental cost-effectiveness ratio
LAGB: laparoscopic adjustable gastric banding
NHS: National Health Service
OAGB: one-anastomosis gastric bypass
OECD: Organization for Economic Cooperation and Development
PICOS: population, intervention, comparator, outcomes, study design
PRESS: Peer Review of Electronic Search Strategies
PRISMA: Preferred Reporting Items for Systematic Reviews and Meta-Analyses
PRISMA-P: Preferred Reporting Items for Systematic Review and Meta-Analysis Protocols
PROSPERO: International Prospective Register of Systematic Reviews
QALY: quality-adjusted life year
RCT: randomized controlled trial
RoB: risk of bias
ROBINS-I: Risk of Bias in Nonrandomized Studies of Interventions
RYGB: Roux-en-Y gastric bypass
SG: sleeve gastrectomy
SWiM: Synthesis Without Meta-Analysis
