# Supplementary material for: Clinical, Economic, and System-Level Outcomes of Bariatric Surgery in Adults: Protocol for a Systematic Review
Source: JMIR Res Protoc. 2026 Oct 19;15:e98976. doi: 10.2196/98976 (PMC13679864; doi:10.2196/98976)
Supplement: Supplementary file 1 [file resprot-v15-e98976-s001.pdf]

PRISMA-P (Preferred Reporting Items for Systematic review and Meta-Analysis Protocols) 2015 Checklist

Recommended items to address in a systematic review protocol*

Section & Topic	Item No.	Checklist Item	Location where item is reported
**ADMINISTRATIVE INFO**			
**Title**			
Identification	1a	Identify the report as a protocol of a systematic review	Title page, p 1 - the title identifies the report as a protocol for a systematic review; also Abstract, p 2, and Results, p 29.
Update	1b	If the protocol is for an update of a previous systematic review, identify as such	Not applicable - this is a protocol for a new systematic review, not an update of a previous review.
**Registration**			
Registration	2	If registered, provide the name of the registry (such as PROSPERO) and registration number	Abstract (Trial Registration), p 3; Methods - Protocol and Registration, p 8; Results, p 29. PROSPERO, registration number CRD420261351022, registered 15 April 2026.
**Authors**			
Contact	3a	Provide name, institutional affiliation, e-mail address of all protocol authors; provide physical mailing address of corresponding author	Title page, p 1 - names, ORCID identifiers, institutional affiliations and e-mail addresses of all three authors, and the mailing address of the corresponding author.
Contributions	3b	Describe contributions of protocol authors and identify the guarantor of the review	Authors' Contributions, p 35 - contributions of MB, MR and MPS; MB is identified as the guarantor of the review.
**Amendments**			
Amendments	4	If the protocol represents an amendment of a previously completed or published protocol, identify as such and list changes; otherwise, state plan for documenting important protocol amendments	Methods - Protocol and Registration, p 8 - amendments will be documented, dated and reported transparently in the final review manuscript; also Dissemination Plan, p 34. This protocol is not an amendment of a previously completed or published protocol.
**Support**			
Sources	5a	Indicate sources of financial or other support for the review	Funding, p 35 - no financial support was received for this work; Acknowledgments, p 35 - institutional support from the Medical University of Gdańsk.
Sponsor	5b	Provide name for the review funder and/or sponsor	Funding, p 35 - not applicable; the review has no external funder or sponsor.

Role of Sponsor or Funder	5c	Describe roles of funder(s), sponsor(s), and/or institution(s), if any, in developing the protocol	Not applicable - no funder or sponsor, and therefore no role in developing the protocol (Funding, p 35; Conflicts of Interest, p 36).
**INTRODUCTION**			
**Rationale**			
Rationale	6	Describe the rationale for the review in the context of what is already known	Introduction, p 4-7: Burden of Obesity (p 4), Role of Bariatric Surgery (p 4-5), Clinical, Economic, and System-Level Relevance (p 5-6) and Rationale for the Review (p 6-7), which states the gap addressed - existing reviews largely predate 2020 and rarely integrate the clinical, economic and system-level domains.
**Objectives**			
Objectives	7	Provide an explicit statement of the question(s) the review will address with reference to participants, interventions, comparators, and outcomes (PICO)	Introduction - Objectives and Review Questions, p 7-8; Abstract - Objective, p 2. Primary objective and three review questions stated in PICO terms; full PICOS framework in Table 1, p 10-11.
**METHODS**			
**Eligibility Criteria**			
Eligibility criteria	8	Specify the study characteristics (such as PICO, study design, setting, time frame) and report characteristics (such as years considered, language, publication status) to be used as criteria for eligibility for the review	Methods - Eligibility Criteria, p 8-9, and Table 1 (PICOS), p 10-11 - population, intervention (primary procedures only; revisional/conversion excluded as the index intervention), comparators, outcomes, eligible and excluded designs, setting, publication window 1 January 2020 - 31 December 2025, English language, full-text peer-reviewed articles.
**Information Sources**			
Information sources	9	Describe all intended information sources (such as electronic databases, contact with study authors, trial registers or other grey literature sources) with planned dates of coverage	Methods - Information Sources, p 11-12 - MEDLINE (via PubMed), Embase, Scopus and Web of Science, with reasons given for not searching Cochrane CENTRAL, EconLit, NHS EED and trial registries; date coverage 1 January 2020 - 31 December 2025 (Table 1, p 11); searches run on 1 August 2026 (Results, p 29). Contact with study authors: Data Extraction, p 19.
**Search Strategy**			

Search strategy	10	Present draft of search strategy to be used for at least one electronic database, including planned limits, such that it could be repeated	Methods - Search Strategy, p 12-18 - dual-track (economic and system-level) block structure described on p 12-13, with complete database-specific strategies and all limits reproduced verbatim for Scopus, MEDLINE/PubMed, Embase and Web of Science, p 13-18.
**Study Records**			
Data management	11a	Describe the mechanism(s) that will be used to manage records and data throughout the review	Methods - Study Selection, p 18 - records imported into Zotero, duplicates removed using an automatic tool with manual verification; Methods - Data Extraction, p 19 - standardised extraction form in spreadsheet software.
Selection process	11b	State the process that will be used for selecting studies (such as two independent reviewers) through each phase of the review (that is, screening, eligibility and inclusion in meta-analysis)	Methods - Study Selection, p 18-19 - two-stage screening (title/abstract, then full text) with pre-defined exclusion categories; single primary reviewer with at least 20% of records independently duplicated at both stages, a calibration tranche screened in duplicate, disagreements resolved by discussion and third-reviewer adjudication.
Data collection process	11c	Describe planned method of extracting data from reports (such as piloting forms, done independently, in duplicate), any processes for obtaining and confirming data from investigators	Methods - Data Extraction, p 19 - standardised form piloted on five studies; extraction primarily by one reviewer with at least 20% of included studies independently re-extracted and discrepancies resolved by consensus; corresponding authors contacted by e-mail (up to two attempts over four weeks) where data are missing or unclear.
**Data Items**			
Data items	12	List and define all variables for which data will be sought (such as PICO items, funding sources), any pre-planned data assumptions and simplifications	Methods - Data Extraction, p 19, and Table 2, p 20-21 - study characteristics, population, intervention, comparator, follow-up, and clinical, economic and system-level outcomes. Pre-planned assumptions: prespecified hierarchy for establishing an unstated price year (p 27) and use of author-reported definitions for system-level indicators (p 8-9).

**Outcomes and Prioritization**			
Outcomes and prioritization	13	List and define all outcomes for which data will be sought, including prioritization of main and additional outcomes, with rationale	Methods - Eligibility Criteria, p 8-9 (clinical and economic outcomes primary, system-level resource use exploratory, with rationale); Table 2, p 20-21; Data Synthesis, p 25 - priority outcomes prespecified: %TWL at 12 and 24 months, remission of type 2 diabetes, overall and severe (Clavien-Dindo ≥III) complications, and cost per QALY gained.
**Risk of Bias**			
Risk of bias in individual studies	14	Describe anticipated methods for assessing risk of bias of individual studies, including whether this will be done at the outcome or study level, or both; state how this information will be used in data synthesis	Methods - Risk of Bias and Quality Assessment, p 22-23, and Table 3, p 23 - RoB 2 for RCTs (overall judgement per outcome), ROBINS-I for non-randomised studies (per study), CHEERS 2022 for reporting completeness and CHEC for methodological quality of economic evaluations, with at least 20% of assessments independently validated. Use in synthesis: flagging of high/critical risk and low-quality studies, narrative sensitivity analysis, and direct input to the GRADE risk-of-bias domain (p 23; p 29).
**Data Synthesis**			
Data synthesis	15a	Describe criteria under which study data will be quantitatively synthesised	Methods - Data Synthesis, p 25-26 - quantitative synthesis is not planned a priori; meta-analysis will be considered only where the prespecified minimum comparability standard is met (same priority outcome, comparable timepoint, definitions, population and procedure category) and effect estimates and variance are reported or derivable, with at least three studies treated as necessary but not sufficient.
	15b	If data are appropriate for quantitative synthesis, describe planned summary measures, methods of handling data and methods of combining data from studies, including any planned exploration of consistency (such as I^2, Kendall's τ)	Methods - Data Synthesis, p 25-26 - random-effects models, heterogeneity quantified using I^2 and τ^2, results presented in forest plots with caveats. Handling of cost data, p 26-28 - extraction in the original currency and price year, inflation within currency using GDP deflators and conversion by purchasing power parities to euros at

			target price year 2024.
	15c	Describe any proposed additional analyses (such as sensitivity or subgroup analyses, meta-regression)	Methods - Data Synthesis, p 25-26 and p 28 - prespecified subgroups (procedure type; follow-up window ≤12, 13-36 and >36 months; healthcare setting or geographical region), narrative sensitivity analysis based on risk of bias and methodological quality (p 23), and one prespecified sensitivity analysis repeating the cost conversion with the alternative purchasing power parity series. Meta-regression is not planned.
	15d	If quantitative synthesis is not appropriate, describe the type of summary planned	Methods - Data Synthesis, p 24-25 - narrative synthesis as the primary method, conducted in line with established narrative-synthesis guidance and reported according to SWiM, with evidence tables grouped by outcome domain and design, exploration of variation by procedure type, region and study quality, and a concluding cross-domain narrative examining coherence and trade-offs.
**Meta-bias(es)**			
Meta-bias(es)	16	Specify any planned assessment of meta-bias(es) (such as publication bias across studies, selective reporting within studies)	Methods - Data Synthesis, p 28-29 - publication bias and outcome reporting bias assessed separately: funnel plots and Egger's test only if a meta-analysis with at least ten studies per outcome is performed, otherwise qualitative appraisal of gaps and imbalances; outcome reporting bias assessed within RoB 2 and ROBINS-I and against protocols or registrations where available.
**Confidence in Evidence**			
Confidence in cumulative evidence	17	Describe how the strength of the body of evidence will be assessed (such as GRADE)	Methods - Certainty of Evidence, p 29 - GRADE applied to the cumulative evidence for each priority outcome across risk of bias, inconsistency, indirectness, imprecision and publication bias, rated high, moderate, low or very low, with summary-of-findings tables; for economic outcomes certainty is informed jointly by CHEERS 2022 (reporting) and CHEC (methodological

			quality).

*It is strongly recommended that this checklist be read in conjunction with the PRISMA-P Explanation and Elaboration for important clarification on the items. Amendments to a review protocol should be tracked and dated. The copyright for PRISMA-P (including checklist) is held by the PRISMA-P Group and is distributed under a Creative Commons Attribution Licence 4.0.

From: *Shamseer L, Moher D, Clarke M, Ghersi D, Liberati A, Petticrew M, Shekelle P, Stewart L, PRISMA-P Group. Preferred reporting items for systematic review and meta-analysis protocols (PRISMA-P) 2015: elaboration and explanation. BMJ. 2015 Jan 2;349(jan02 1):g7647.*
